# Lipid-lowering effect and oral transport characteristics study of curculigoside

**DOI:** 10.3389/fcvm.2024.1426379

**Published:** 2024-07-02

**Authors:** Aiping Wang, Jie Ning, Lu Zhao, Renjie Xu

**Affiliations:** ^1^ Shaoxing Maternity and Child Health Care Hospital, Shaoxing, China; ^2^ Jiangsu Key Laboratory of New Drug Research and Clinical Pharmacy, Xuzhou Medical University, Xuzhou, China

**Keywords:** curculigoside, transport, lipid-lowering, mechanism, natural products

## Abstract

**Introduction:**

The incidence of metabolic disorders during pregnancy is increasing year by year, with diseases including hypertension and hyperlipidemia. Statins are the primary drugs for treating hyperlipidemia or atherosclerosis, yet some patients remain unresponsive to them, and pregnant women are prohibited from taking statins. Curculigoside is the major biologically active natural product present in *Curculigo orchioides*.

**Methods:**

In this study, A high-fat mice model was developed to study the lipid-lowering effect of curculigoside. Using intestinal Caco-2 cell monolayer, the curculigoside transport properties at two temperatures and possible transporters were systemically studied.

**Results:**

Curculigoside at concentrations used during the experiments have no toxic effect to Caco-2 cells. The curculigoside transfer from the apical to the basolateral side was strongly influenced by temperature. P-glycoprotein, breast cancer resistance protein, and efflux transporters are crucial components of the human intestinal cell line Caco-2. The curculigoside can significantly affect the contents of total cholesterol, triglycerides, high-density lipoprotein cholesterol, and low-density lipoprotein cholesterol in mice.

**Discussion:**

The transport properties and potential mechanism of curculigoside offer valuable insights for the design of development of hypolipidemic drugs like anti-atherosclerotic drugs and also be helpful to the further study of the pharmacological activity of curculigoside.

## 1 Introduction

Large- and medium-sized arteries are affected by atherosclerosis, which primarily affects the coronary arteries. High low-density lipoprotein (LDL) levels, total cholesterol (TC) levels, triglycerides (TG) levels, hypertension, smoking, diabetes, and a family history of the condition are conventional risk factors for atherosclerosis (1). In pregnant women, high LDL levels may affect fetal development and increase the risk of gestational diabetes or hypertension. Lipid-reducing medications known as statins have become the most effective treatments and preventative measures for atherosclerosis. However, there are still unanswered concerns about the pathophysiology of atherosclerosis, which emphasizes the need for innovative treatment approaches to treat those for whom statins are ineffective. The proportion of suboptimal efficacy in treating cardiovascular diseases with statins is relatively high. This suboptimal efficacy typically refers to the failure to achieve target levels of low-density lipoprotein cholesterol (LDL-C). According to various studies and clinical data, approximately 20%–30% of cardiovascular patients worldwide fail to reach their LDL-C target levels while on statin therapy (2). High cholesterol or high triglycerides in pregnant women may increase the risk of pregnancy complications such as gestational hypertension and gestational diabetes. Elevated blood lipid levels can also negatively affect fetal development, increasing the risk of preterm birth and low birth weight. Statins are widely considered unsuitable for use in pregnant women. Studies have shown that these drugs may have potential teratogenic effects on the fetus, especially during early pregnancy. Statins can cross the placental barrier and may negatively impact fetal development. Therefore, finding safe and effective lipid-lowering medications that can be used during pregnancy has significant potential. Fortunately, a variety of natural product derivatives and extracts have anti-atherogenic activity, indicating viable treatment possibilities for atherosclerosis especially in pregnant women (3).

*Curculigo orchioides Gaertn* is a member of the Amaryllidaceae family, which is extensively found in China, Australia, Malay and India. The traditional Chinese medicinal system makes extensive use of the rhizome as a significant herbal remedy (4). Curculigoside, a phenolic glycoside compound, is the major biologically active ingredient present in *Curculigo orchioides*. Previous studies reported that curculigoside shows wide spread pharmacological activities including antioxidant (2, 5), angiogenesis (6), neuroprotection (7) and anti-osteoporosis (8). Some earlier studies showed that curculigoside can protect endothelial cells against oxidative injury induced by H_2_O_2_, suggesting that this compound may constitute a promising intervention against cardiovascular disorders. As a traditional Chinese medicine, curculigoside may be used to create oral administration medications or dietary supplements to prevent cardiovascular (9). The absorption properties of oral medicine like curculigoside play crucial roles in plasma drug concentration and therapeutic efficacies (10).

The intestinal mucosa serves as the primary barrier to oral absorption, and the lipophilicity and solubility characteristics, as well as the molecular size, charge, and hydrogen bond potential of oral medications, primarily influence the transporters/carriers found in the enterocyte's apical and basolateral plasma membranes (11). Additionally, concentration gradient induced passive diffusion serves as the primary drug absorption transport mechanism across the intestinal epithelium (12). The intestinal Caco-2 cell monolayer may be the most suitable model to study oral drug absorption since the human colon cancer cell line Caco-2 may develop into monolayers with numerous functions in the epithelium of the small intestine villus (13). In this work, we examined the lipid-lowering effect of curculigoside and how temperature and associated transporters affect curculigoside trafficking.

## 2 Materials and methods

### 2.1 Chemicals and biological materials

**Figure 1:**
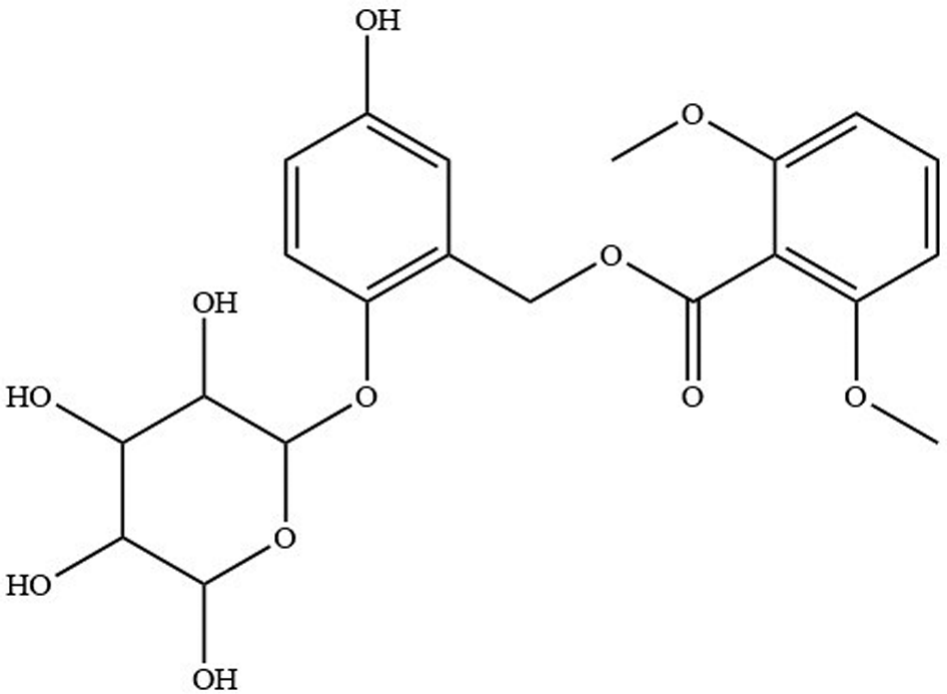
The chemical structure of curculigoside.

Curculigoside (Figure 1) was isolated from *Curculigo orchioides* (14). Gibco Laboratory (Invitrogen Co, Grand Island, NY, USA) supplied Hank's balanced salt solution (HBSS), fetal bovine serum, and antibiotic solutions. Aladdin Reagent (Shanghai, China) provided the following products: Curculigine A, Verapamil, Indomethacin, MK571, Sodium vanadate, Benzbromarone and Cimetidine. The other substances were all analytically graded.

### 2.2 Cell culture

The Chinese Academy of Sciences' standard Culture Collections Committee cell library (Shanghai, China) provided the Caco-2 cells. 75 cm^2^ flasks containing DMEM were used to cultivate the cells. The temperature was maintained at 37 °C in an air that contained 5% CO_2_ (15).

### 2.3 Cytotoxicity measurement

Curculigoside's cytotoxicity was assessed using the MTT test. Caco-2 cells were seeded in 96-well plates at a seeding density of 2 × 10^4^ cells/well in 200 μl medium and cultured at 37 °C in an atmosphere of 5% CO_2_. Following incubation for 24 and 48 h at 37 °C, the cells were grown to 70% confluence. The culture medium in 96-well plates was then replaced with HBSS containing escalating concentrations of curculigoside (1–100 μM), and cell proliferation was assessed using an MTT assay. Following the formation of each cell group, 20 μl of diluted MTT solution (5 mg/ml) in PBS was applied to each well. Following a 4-h incubation period, each well received 200 μl of DMSO after the MTT media was withdrawn. The plates were then scanned on a microplate reader at a wavelength of 490 nm (16) after shaking for 10 min at room temperature. Data were expressed as cell viability (%) of controls that were not exposed to curculigoside.

### 2.4 Transport experiments

In the transport experiments, transwell inserts were used to seed cells at a density of 2.5 × 10^5^ cells/cm^2^. The inserts were then placed in 12-well plates (17). After seeding, Caco-2 cells were allowed to grow and differentiate for 18–20 days in 12-well plastic plates and the medium were changed every 2 or 3 days (18). The monolayer's integrity was monitored using the transepithelial electrical resistance (TEER), which was measured using a Millicell-ERS electrode. In the current investigation, a monolayer with TEER measured between 500 and 750 Ω cm^2^ that was collected before to and following the conclusion of transport tests was used (19).

The monolayers were twice washed with prewarmed HBSS solution before to the experiments (pH 7.4) and subsequently preincubated (37 °C, 10 min) (20). Next, apical (A, 0.5 ml) or basolateral (B, 1.5 ml) doses of curculigoside solutions (5 and 20 μM) were introduced, while the receiving chamber held the equivalent volume of transport medium (21). The Caco-2 cell monolayers were cultured at 37 °C, and samples (50 μl) were removed from the receiving chamber from 15 to 90 min. The same volume of HBSS (37 °C) was then immediately replaced (15). The curculigoside (20 μM) transit at 4 °C and 37 °C was assessed in the A to B direction to look into the impact of temperature. This is how the apparent permeability coefficient (P_app_) was determined:

$$P_{\mathrm{app}}\;=\;(\mathrm{dQ}/\mathrm{dt})\;({1/\mathrm{AC}}_{0})$$

where A is the surface area of the membrane, C_0_ is the donor chamber's starting curculigoside concentration, and dQ/dt represents the time-dependent change in concentration on the receiving side. The quantification of curculigoside transport across Caco-2 cell monolayers was performed using P_app_AB in A to B and P_app_BA in the B to A.

Several transporter inhibitors to determine the transport characteristics of curculigoside: Verapamil (100 μM) was used to inhibit the efflux of P-glycoprotein (P-gp) (22); indomethacin (200 μM), MK 571(100 μM) and benzbromarone (50 μM) were used as multi-drug resistance associate protein (MRPs) inhibitors (22–25); Apigenin (25 μM) was used to inhibit the efflux of breast cancer resistance protein (BCRP); cimetidine (50 μM) and Sodium vanadate (50 μM) were used on Na/K-ATP_ase_ and organic anion transporter (OAT) respectively (26, 27). Before taking Samples, the inhibitors were added on the A and the B, and preincubated the monolayers at 37 °C for 15 min.

### 2.5 Analytical methods

Before analysis, 10 μl internal standard (IS, curculigine A, 10 ng/ml) and 50 μl collected sample was added in a 1.5 ml centrifuge tube, 190 μl of acetonitrile (4:1, v/v) was then added, and the mixture was vortexed for a minute. Following a 10-min centrifugation at 14,000 rpm, a 2-μl aliquot of the supernatant was introduced into the UPLC-MS/MS apparatus.

The following gradient elutions were used: 10% A (0–1.5 min), 40%–95% A (1.5–1.6 min), 95% A (1.6–2.7 min) and 95%–10% A (2.7–2.8 min), 10% A (2.8–4.0 min). The flow rate was 0.4 ml/min at 20 °C (28). The ESI needle voltage was set to −5,500 V and the nebulizer temperature was set to 550 °C. The transition from precursor ion [M + Na]^ +^ at m/z 489.2 to product ion at 205.1 for curculigoside was monitored; to detect curculigine A, the transition from the precursor at m/z 553.2 to the product at 346.9 was monitored.

### 2.6 Animal experiment

Thirty female C57BL/6 mice (8 weeks old, weighing 20 ± 2 g) were obtained from the Laboratory Animal Center of Xuzhou Medical University (Xuzhou, China). All animal experiments obtained approval from the Animal Ethics Committee of Xuzhou Medical University. Atherosclerosis was induced using a pro-atherosclerotic high-fat diet (HFD, 42% fat calories and 0.15% cholesterol) for 16 weeks. During the modeling period, the curculigoside group was administered 100 mg/kg via intragastric administration, while the model group and the control group received normal saline. After an overnight fasting period, 500 μl of peripheral blood was collected from mice in the three groups. The whole blood was left to clot at room temperature for 1 h to obtain serum. Serum levels of TC, TG, HDL cholesterol, and LDL cholesterol were measured.

### 2.7 Statistical analysis

Every data point was presented as mean ± SD. A one-way analysis of variance using IBM SPSS 19.0 (New York, USA) was used to establish statistical significance, with a cutoff point of *p* < 0.05. GraphPad Prism (Windows, version 6.0) was used to draw graphs.

## 3 Results

### 3.1 The result of MTT cytotoxicity assay

**Figure 2:**
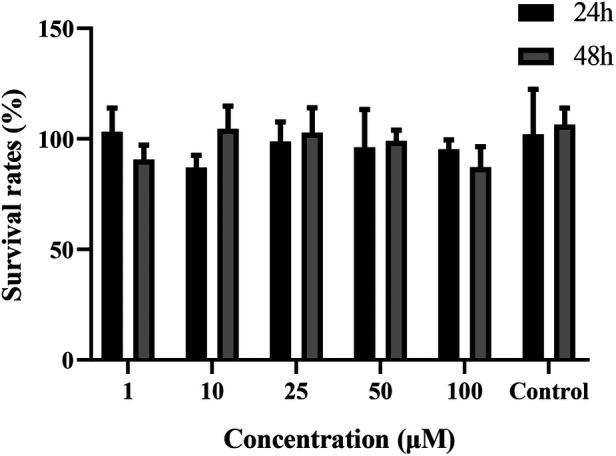
Cytotoxicity of curculigoside. Cytotoxicity of curculigoside was determined by MTT assay. Curculigoside at concentrations used during the experiments have no toxic effect to Caco-2 cells.

The effects of curculigoside on Caco-2 cells are shown in Figure 2. Between the groups receiving curculigoside therapy, no statistically significant variations were observed in the viability of the cells (1–100 μM, for 24 or 48 h) and control group. Curculigoside at concentrations used during the experiments have no toxic effect to Caco-2 cells.

### 3.2 Curculigoside transport across Caco-2 cell monolayers over time

**Figure 3:**
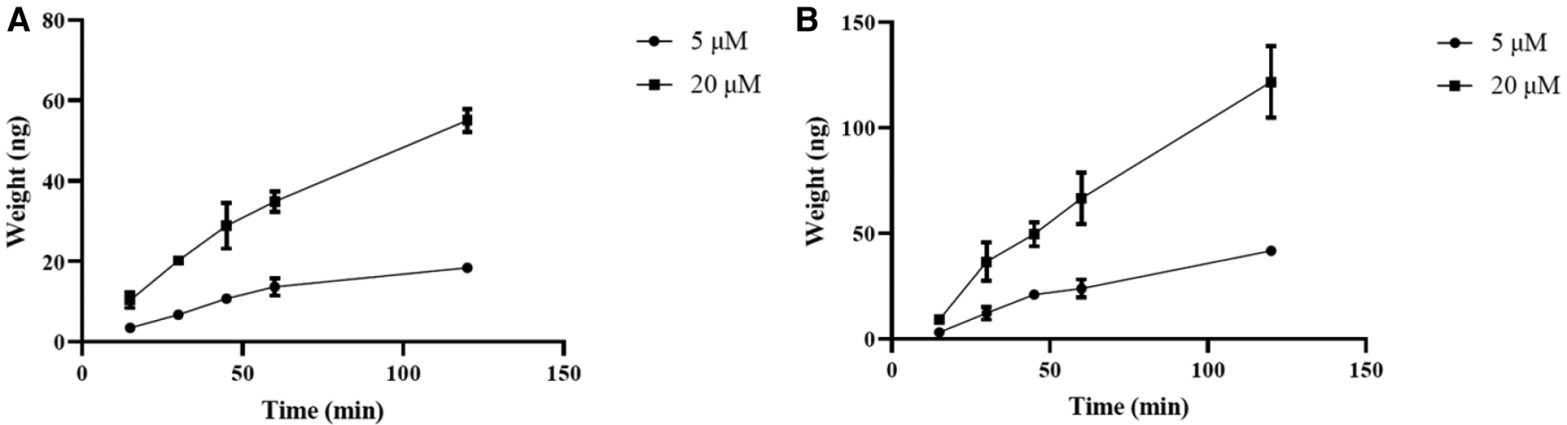
Curculigoside transport across Caco-2 cell monolayers over time. The Caco-2 cell monolayers were twice washed with HBSS media (pH 7.4) before to the experiment. Curculigoside (5 or 20 μM) was incubated on the AP or BL side of the Caco-2 cell monolayers. Over the course of 90 min, curculigoside's transcellular transport rose linearly from the A to B side (**A**) and from the B to A side (**B**). The mean ± SD is used to represent the results (n = 3).

Curculigoside's transcellular transport rose linearly throughout a 90-min period, as illustrated in Figure 2, both from the A to B (Figure 3A) and from the B to A side (Figure 3B).

### 3.3 The effect of temperature on curculigoside transport

**Figure 4:**
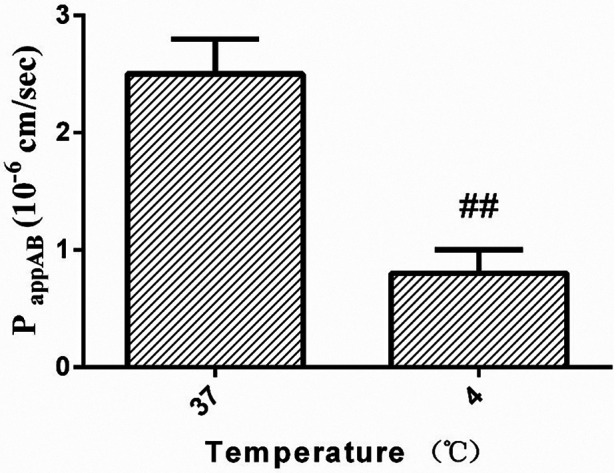
The effect of temperature on curculigoside transport. The Caco-2 cell monolayers were twice washed with HBSS media (pH 7.4) before to the tests. The curculigoside (20 μM) transport at 4 °C and 37 °C was evaluated in the A to B direction. Results are represented as the mean ± SD. (n = 3) ^##^: *P* < 0.01 compared to the control group.

As shown in Figure 4, the temperature had a considerable impact on the curculigoside transfer from A to B side. As the temperature of HBSS medium reduced from 37 °C to 4 °C, the P_app_ AB was changed from (2.5 ± 0.3) × 10^6^ cm/s to (0.8 ± 0.2) × 10^6^ cm/s, which might indicate that the movement of curculigoside was energy-dependent due to the fact that a reduction in temperature could inhibit cellular metabolism (29).

### 3.4 The effects of transporters on curculigoside transport

**Figure 5:**
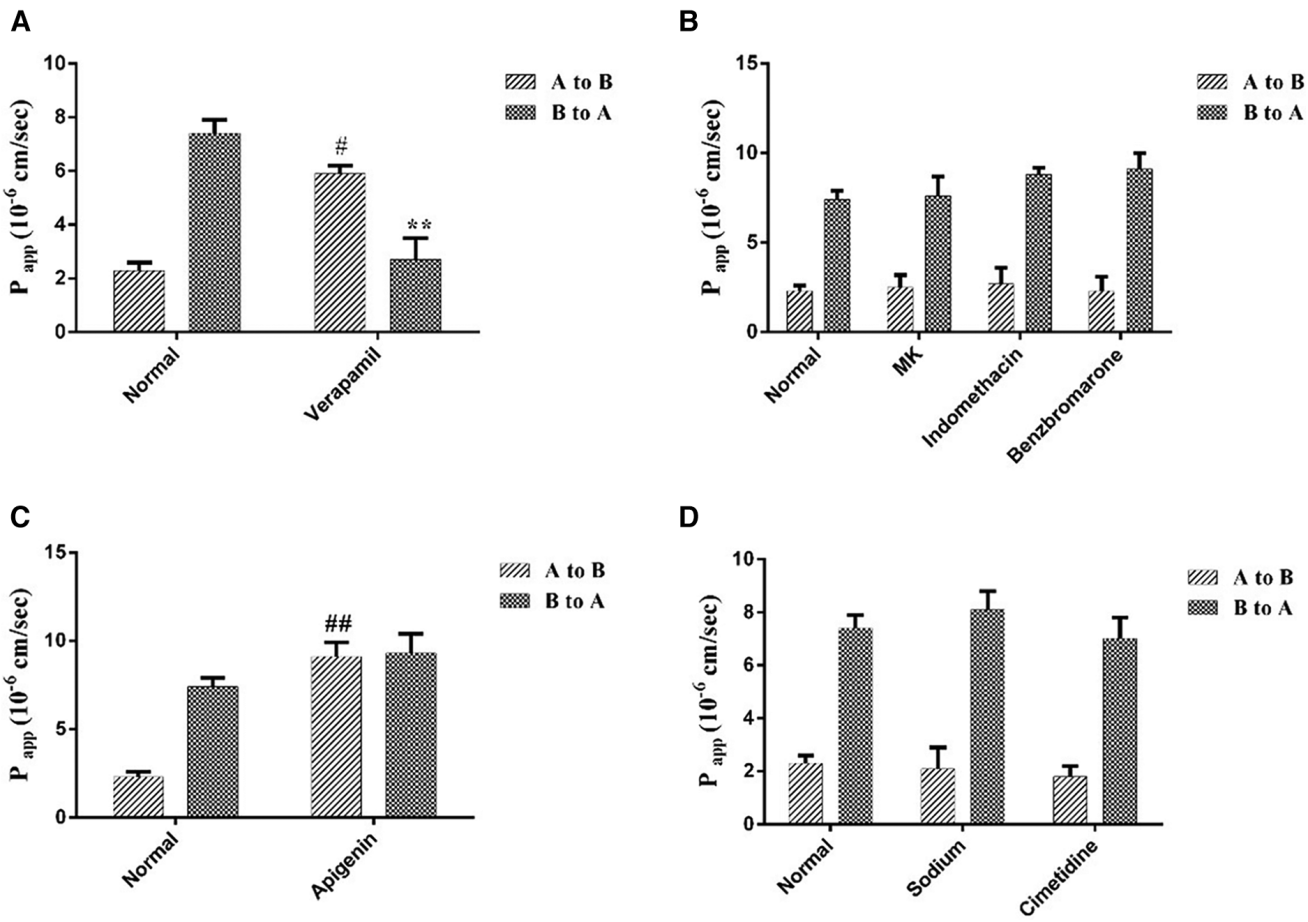
The effects of transporters on curculigoside transport. The Caco-2 cell monolayers were twice washed with HBSS media (pH 7.4) before to the experiment. Verapamil was used to inhibit the efflux of P-gp (**A**); MK 571, benzbromarone and indomethacin were used asMRPs inhibitors (**B**); Apigenin was used to inhibit the efflux of BCRP (**C**); The selectivity of Na/K-ATPase and OAT were evaluated by Sodium vanadate and cimetidine (**D**). Results are represented as the mean ± SD. (n = 3). #: *P* < 0.05 compared to the control group (A to B); ##: *P* < 0.01 compared to the control group (A to B); **: *P* < 0.01 compared to the control group (B to A).

Different transporter inhibitors brought different degree effects on curculigoside transport. Verapamil, a selective for P-gp inhibitor, significantly improved the P_app_ AB from (2.3 ± 0.3) × 10^6^ cm/s to (5.9 ± 0.3) × 10^6^ cm/s, as well as reduced the P_app_ BA from (7.4 ± 0.5) × 10^6^ cm/s to (2.7 ± 0.8) × 10^6^ cm/s (Figure 5A). However, the MRPs inhibitors, MK 571, benzbromarone and indomethacin had on significant effects on curculigoside transport (Figure 5B). These findings suggested that curculigoside was not transported transcellularly by the MRPs transporters. As shown in Figure 5C, the P_app_ AB of BCRP inhibitor (apigenin) treated group was increased almost 6.0-fold, implying that BCRP transporters were probably the most important influx transporters on the curculigoside secretion. In addition, sodium vanadate and cimetidine (Figure 5D) showed marginal effect on P_app_ AB or P_app_ BA of curculigoside transport, implying that Na/K-ATPase and OAT were almost not involved in curculigoside transport.

### 3.5 The effects of curculigoside on serum parameters of atherosclerotic mice

**Figure 6:**
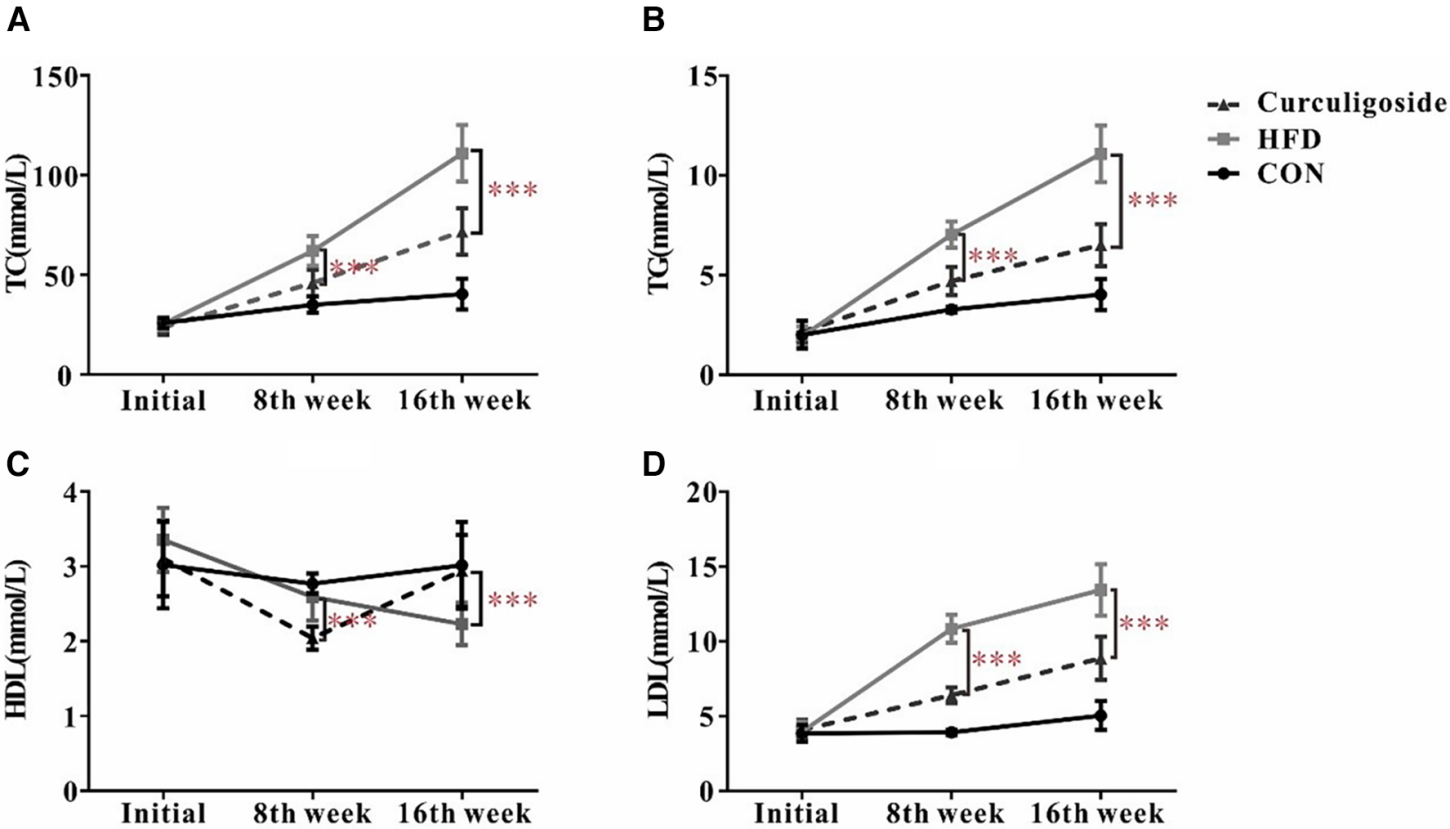
Effects of curculigoside on serum lipids. TC (**A**), TG (**B**), HDL (**C**), and LDL (**D**) from the serum of C57BL/6 mice measured at 8th week and 16th week. Plots are expressed as mean; error bars: SD; *n* value: 10; *: *P* < 0.05, ***: *P* < 0.001.

Given the well-established role of hyperlipidemia in promoting atherosclerosis, we focused on assessing the lipid profile of mice. The serum levels of TC (Nanjing Jiancheng, # A111-1-1, Nanjing, China), TG (Nanjing Jiancheng, # A110-1-1, Nanjing, China), HDL cholesterol (Nanjing Jiancheng, # A112-1-1, Nanjing, China), and LDL cholesterol (Nanjing Jiancheng, # A113-1-1, Nanjing, China) were measured. As depicted in Figure 6, the HFD diet markedly increased levels of TC, TG, HDL and LDL compared to the control group. At the same time, significant differences in TC, TG, HDL and LDL were observed subsequent to the administration of curculigoside intervention at the 8th week and 16th week.

## 4 Discussion

Despite treatment advancements, atherosclerosis, characterized by lipid accumulation and fatty plaque formation in blood vessels, remains a chronic multifactorial vascular disease and the primary cause of cardiovascular disease, with high levels of morbidity and mortality (30). Statins have emerged as preferred medications for lipid-lowering therapy and antiatherosclerosis in clinical practice, significantly reducing plasma cholesterol levels to slow atherosclerosis progression. However, due to the complexity of the pathology of atherosclerosis, patients who are resistant to statins require new medications. Many natural products or components of traditional Chinese medicine are considered safe for use in pregnant women. Since statins cannot be used by pregnant women, researching natural products with lipid-lowering effects is considered a potential alternative to statins. However, natural products, especially those traditionally ingested orally, may face challenges like poor absorption and unclear mechanisms of action. Therefore, a series of experiments is essential to investigate absorption and the mechanisms of their effects.

The oral absorption characteristics of a drug are part of its pharmacokinetic profile, mainly affecting the absorption of orally administered drugs into the bloodstream and the concentration levels that can ultimately reach target organs to exert therapeutic effects. Experimental results have shown that many components of traditional Chinese medicine, including curculigoside, have relatively good oral absorption, which aligns with the traditional method of decoction administration in Chinese medicine. In this experiment, the lipid-lowering effects of orally administered curculigoside were preliminarily explored, and the initial experimental results demonstrated that curculigoside can exert its therapeutic effects through oral administration. Statins are considered to have good absorption profiles in pharmacokinetic evaluations when administered orally. Most statins are rapidly absorbed from the gastrointestinal tract, although their absolute bioavailability can vary due to factors such as first-pass metabolism. Despite this, the effectiveness of statins in lowering cholesterol levels and their widespread clinical use indicate that their oral absorption is sufficient to achieve therapeutic blood concentrations. Compared to statin drugs, curculigoside has poorer absorption in the small intestine. Many components of traditional Chinese medicine face this issue, yet it does not seem to affect their therapeutic efficacy. However, further research is needed to explore whether these absorption differences can impact the efficacy and dosing, particularly in the context of lipid-lowering effects.

Research has shown that Curculigoside significantly enhanced cell viability, reduced cell apoptosis and LDH activity, decreased infarct size and myocardial apoptosis in both H9c2 cells and isolated rat hearts. Furthermore, it downregulated the expression of cytochrome c, apoptotic protease activating factor-1, cleaved caspase-9, and cleaved caspase-3 (31). Curculigoside can mitigate hepatic ischemia-reperfusion injury by activating the Nrf-2/HO-1 pathway, which exerts antioxidant effects, suppresses inflammatory cell infiltration, and inhibits the secretion of pro-inflammatory cytokines, thereby alleviating hepatic ischemia-reperfusion injury (32). Inflammatory conditions, oxidative stress, and endothelial dysfunction are fundamental mechanisms in the development of cardiovascular disease, especially atherosclerosis, wherein oxidative stress plays a pivotal role in its pathogenesis (33). Therefore, Curculigoside may potentially lower lipid levels in atherosclerotic mice through its antioxidative and anti-inflammatory effects. However, further research is needed to elucidate its mechanisms of action on vascular morphology and protective effects. The primary aim of this study is to explore the intestinal absorption mechanism of curculigoside, building upon its established anti-atherosclerotic effects. This research endeavors to offer insights for future investigations into its mechanisms against atherosclerosis and pharmacokinetic properties. However, it is important to acknowledge the limitations of this study. Due to the imperfect experimental design, the evidence supporting the success of the modeling was not adequately reflected. Additionally, there was a lack of positive control drugs for comparison with curculigoside. These issues will be addressed in our subsequent research. Caco-2 monolayers can be used to choose pharmaceuticals with superior passive absorption characteristics from an assortment of pharmacologically active compounds and to identify drugs with possible absorption issues because drug transport in these monolayers is a good practical way to predict the movement of compounds as intestinal drug transport *in vivo* (34). While the physiologically active component of *Curculigo orchioides* is curculigoside, intestinal drug absorption could be a challenge for the development of oral medications or dietary supplements intended to prevent diseases. The A or B membrane of enterocytes contains ATP-binding cassette (ABC) transporters, which possess the capability to promote absorption into the bloodstream or excretion back into the intestinal lumen (35). In this study, we used ABC transporters containing P-gp, BCRP and MRPs, Na/K-ATPase and OAT inhibitors to predict the transport characteristics of curculigoside in the human intestinal cell line Caco-2. The curculigoside transport was mainly affected by P-gp and BCRP.

P-gp transports medicines out of cells, which is linked to multidrug resistance. The bioavailability of many medications is significantly impacted by P-gp's efflux transport (36). In addition, many natural compounds absorption was limited by the P-gp efflux transport. Isorhamnetin, a flavonoid existed in various traditional herbal medicine and plant-based food. In Caco-2 cell monolayers, the treatment of two P-gp inhibitors, Verapamil and nifedipine, dramatically improved the absorption of isorhamnetin (36). Many naturally occurring substances, including biochanin A and silymarin, can block P-gp-mediated efflux in Caco-2 cells, and using P-gp substrate medications in conjunction may improve absorption and bioavailability (37).

MRPs are another class of ABC transporters associated with drug efflux in Caco-2 cells. The MRP2 inhibitor MK571 inhibits the efflux of morroniside and sweroside in Caco-2 cells (38). In our study, the MRPs inhibitors, MK 571, benzbromarone and indomethacin had on significant effects on curculigoside efflux, which proved that MRPs were not involved in the absorption process of curculigoside.

BCRP is an efflux transporter with a broad substrate specificity and high capacity (39). An excellent model for examining intestinal BCRP-mediated transport is the Caco-2 cell (31). Curculigoside's efflux into the apical compartment of the monolayers was reduced upon the addition of the BCRP inhibitor, apigenin. In conclusion, the pattern of inhibition by various ABC transporter inhibitors indicates that BCRP is primarily involved in the apical efflux of curculigoside. During the experiments, Sodium vanadate and cimetidine had on significant effects on curculigoside P_app_AB or P_app_BA of curculigoside transport. Therefore, influx transporters (the selectivity of Na/K-ATPase and OAT) were not involved in curculigoside transport.

## 5 Conclusion

In conclusion, our study demonstrated the lipid-lowering effect of curculigoside in high-fat mice. We also investigated the transport pathway of curculigoside in Caco-2 cells, emphasizing the importance of P-gp, BCRP, and efflux transporters in this human intestinal cell line. The transport properties and potential mechanism of curculigoside offer valuable insights for the design of development of anti-atherosclerotic drugs and also be helpful to the further study of the pharmacological activity of curculigoside.

## Data Availability

The original contributions presented in the study are included in the article/Supplementary Material, further inquiries can be directed to the corresponding authors.
